# Serological Responses up to 9 Months following COVID-19 mRNA Vaccination in Residents and Health-Care Workers of Long-Term Care Facilities: A Multicenter Prospective Cohort Study in Northern Italy

**DOI:** 10.3390/vaccines10122183

**Published:** 2022-12-19

**Authors:** Costanza Vicentini, Carla Maria Zotti, Alessandro Roberto Cornio, Jacopo Garlasco, Noemi Marengo, Davide Meddis, Savina Ditommaso, Monica Giacomuzzi, Gabriele Memoli, Valerio Bordino, Maria Michela Gianino, on behalf of the Collaborating Group

**Affiliations:** Department of Public Health and Paediatrics, University of Turin, Via Santena 5bis, 10126 Torino, Italy

**Keywords:** COVID-19, LTCFs, Italy

## Abstract

Long-term care facilities (LTCFs) were severely affected by COVID-19, in particular in Northern Italy. We aimed to assess antibody responses among residents and healthcare workers (HCWs) of 13 LTCFs through serum samples collected at three time points: prior to, two weeks, and 9 months after receiving Pfizer/BNT162b2 SARS-CoV-2 mRNA vaccine (respectively t0, t1, and t2). IgG antibodies targeted towards the S1 domain of the spike protein were measured, and results were expressed in binding antibody units (BAU/mL). Friedman’s average rank test was performed to compare antibody titres between the three time points. Two logistic regression models were built to identify independent predictors of (1) developing and (2) maintaining a significant antibody response to vaccination, using a previously identified threshold. In total, 534 subjects were enrolled (371 HCWs and 163 residents). The antibody titres at t1 were the highest; at t2, the IgG titres significantly decreased, remaining however 10 times higher compared to titres at t0. Previous infection was the only significant predictor of developing and maintaining a response over threshold in both models. Results of this study provided further insights on the humoral response elicited by vaccination, and on host factors determining variations in its magnitude and kinetics.

## 1. Introduction

Italy was one of the first European countries to be hit by the COVID-19 pandemic, and, as of August 2022, over 20 million SARS-CoV-2 cases and 176,322 related deaths have been reported to the Italian surveillance system [1,2]. Long-term care facilities (LTCFs) have been severely affected by the pandemic, in particular in the North of Italy, adding to the already significant burden of healthcare-associated infections [3,4,5].

Serological investigations can be used to assess previous viral exposure or the presence of an immune response. Since the early stages of the pandemic, the estimated percentage of the population with antibodies against SARS-CoV-2 virus and its variants have gradually increased, both due to viral circulation and to the introduction of safe and effective vaccines. In Italy, the COVID-19 vaccination campaign began in December 2020, and by September 2021, the vaccination coverage among LTCF residents was over 90%, leading to significant reductions in both the occurrence of cases and related deaths [6].

Elderly and frail individuals were initially under-represented in vaccine clinical trials; however, a growing body of literature has been dedicated to studying the immune response among this population [3,7,8]. It is particularly important to study the duration of immunity among elderly individuals, as they are considered at high risk of developing severe COVID-19 infection [9,10], and the immune response to vaccines could be negatively affected by immunosenescence [11].

Results of several studies suggest that antibodies usually last between 3 to 6 months following infection, with one study reporting a duration of up to 11 months [12]. Concerning antibody response following vaccination, anti-receptor-binding domain (RBD) antibodies have been identified several months after vaccination [13]. However, several studies suggest circulating antibody titres following vaccination against SARS-CoV-2 gradually decrease over time [14].

Long-term prospective studies are necessary to investigate the duration and behaviour of antibody levels over time in different populations. Results of these studies are relevant to inform pandemic response strategies, and are of particular importance to ensure that the most vulnerable populations are effectively protected.

This study follows on from two previous serosurveys conducted on residents and healthcare workers (HCWs) of 13 LTCFs in northwestern Italy at two time points: prior to and two weeks after receiving Pfizer/BNT162b2 SARS-CoV-2 mRNA vaccine [15]. The purpose of this multicentre study was to assess antibody responses up to 9 months after vaccination, and to investigate predictors of developing and maintaining a significant response over time.

## 2. Materials and Methods

### 2.1. Study Design, Participants and Data Collection

This study is the final instalment of a multicentric, prospective cohort study designed to evaluate SARS-CoV-2 specific IgG titres prior to and at two time points after receiving a full vaccination cycle with Pfizer/BNT162b2 SARS-CoV-2 mRNA vaccine (two doses of vaccine, 21 days apart). The study protocol was previously described in detail [15]. Briefly, HCWs (doctors, nurses and nursing aides) and residents of 13 LTCFs of the region of Piedmont, in Northern Italy, were invited to participate in the study on a voluntary basis. Participant enrolment was carried out in January 2021, and all participants completed the vaccination cycle between January and March 2021. Serum samples were collected at the following three time points: (t0) prior to vaccination, (t1) two weeks after and (t2) 9 months after completing the vaccination cycle. Data on demographic and clinical characteristics of participants, including age, gender and previous SARS-CoV-2 infections confirmed by reverse-transcription polymerase chain reaction (RT-PCR) testing, were collected at t0. Furthermore, data on the occurrence of breakthrough infections were collected at t1 and t2. 

The research protocol was in accordance with the Declaration of Helsinki and fulfilled the requirements of Italian (Law 2003/196) and European regulations (GDPR EC/2016/679) concerning data protection and privacy. The study was approved by the relevant Institutional Review Boards (Local Health Authorities of Alessandria, Cuneo, and Turin, protocol numbers COV 28/2020, 10077 and 0016945). Informed consent was obtained from all study participants prior to the collection of data and specimens. 

### 2.2. Laboratory Analysis

Laboratory analyses were conducted at the Laboratory of Serology and Microbiology applied to Hygiene of the Department of Public Health and Paediatrics of the University of Turin, as described previously [15]. SARS-CoV-2 IgG antibodies were measured using the EUROIMMUN Anti-SARS-CoV-2 QuantiVac ELISA kit (EUROIMMUN Medizinische Labordiagnostika AG, Lübeck, Germany). The kit detects IgG antibodies using the S1 domain of the spike protein, including the RBD. Results were to be interpreted as negative if lower than 8 relative units (RU)/mL, borderline if between 8 and 11 RU/mL, and positive if ≥11 RU/mL, in line with the instructions of the kit manufacturer. RUs were converted into binding antibody units (BAU) using the conversion factor identified by the WHO International Standard for COVID-19 serological tests (3.2 for EUROIMMUN QuantiVac ELISA IgG) [16].

### 2.3. Statistical Analysis

Descriptive statistics were used to represent demographic and clinical characteristics including antibody titres. Medians and interquartile ranges (IQRs) were used to describe continuous variables, due to non-normal distribution (Shapiro–Wilk test), and categorical variables were reported as numbers and percentages. Statistically significant differences in categorical and continuous variables were investigated using chi-squared and Mann–Whitney U tests, respectively.

Friedman’s average rank test was performed to compare antibody titres between the three time points (t0, t1, t2) among all participants and among participants with vs. without a previous SARS-CoV-2 infection. Mann–Whitney U tests were applied to detect statistical differences in antibody titres between subgroups (HCWs vs. residents of LTCFs, female vs. male, previous SARS-CoV-2 infection < 3 months before vaccination vs. >3 months before vaccination). 

Two logistic regression models were built to identify independent predictors of (1) developing and (2) maintaining a significant antibody response to vaccination, using the threshold identified by Van Praet et al. and Blain et al. (≥1050 arbitrary units, AU/mL, equivalent to 149.1 BAU/mL using a conversion factor of 0.142) [17,18,19]. The following independent variables were considered in both models: age, gender, participant type (HCWs vs. residents) and previous SARS-CoV-2 infection, whereas the considered outcomes were respectively: (1) IgG response at t1 over threshold and (2) IgG response at t2 over threshold. 

The significance level was set at α = 0.05 for all analyses. The statistical software SPSS version 27.0 (IBM, Armonk, NY, USA) was used for all computation and plotting.

## 3. Results

**Figure 1:**
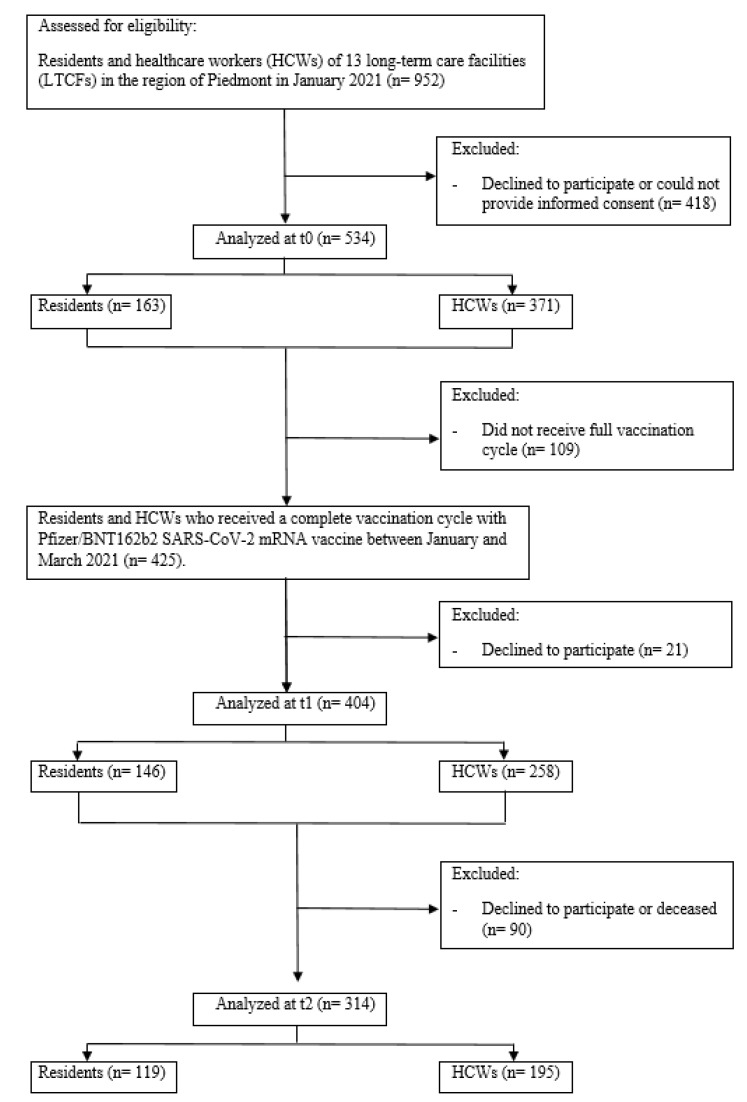
Study flow chart. HCWs: healthcare workers; LTCF: long-term care facility; t0: time period prior to vaccination; t1: two weeks after after completing the vaccination cycle; t2: 9 months after completing the vaccination cycle.

A flow chart of included participants is presented in Figure 1. Considering 952 initially eligible subjects, 534 subjects were enrolled at t0 (371 HCWs and 163 residents of LTCFs). After the beginning of the vaccination campaign, 404 subjects were analysed at t1. Nine months after completing the vaccination cycle, 314 subjects were studied at t2 (195 HCWs and 119 residents). 

Table 1 summarizes characteristics of study participants. Age had a bi-modal distribution, in line with participant type: the median age among HCWs was 47 years (IQR 38–54), and was 86 years (IQR 80–90) among residents. Overall, 25.66% of included participants were over 75 years old. The majority of participants were female, both among HCWs and residents (83.01 and 70.55%, respectively). Over half of study participants had a SARS-CoV-2 infection confirmed by RT-PCR prior to t0; however, the proportion of seropositive individuals at t0 was 37.83%. The IQR of days between RT-PCR testing and t0 was broad, from 47.5 to 262 days. Almost all participants were seropositive two weeks following vaccination, and 94.59% remained positive after nine months. However, while 98% of participants had an IgG titre above the threshold of a significant response at t1, only 27.07% maintained a titre above threshold at t2. No breakthrough infection was reported between vaccination and t2.

**Figure 2:**
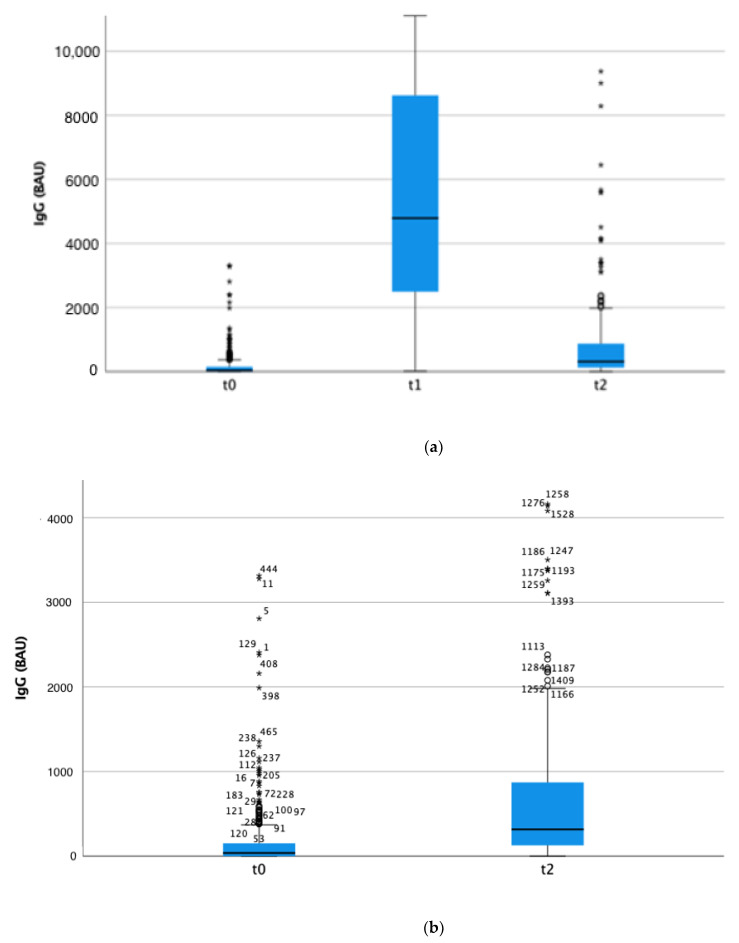
Box plots of anti-SARS-CoV-2 IgG titres (BAU/mL) among all study participants (t0: N = 534, t1: N = 404, t2: N = 314). (**a**) t0, t1 and t2; (**b**) t0 vs. t2. * and circles: outliers.

IgG titres considering all participants were significantly different between the three time points, as shown in Figure 2 (*p* < 0.001 at Friedman’s test). The antibody titres at t1 (after full vaccination cycle) were the highest; at t2, the IgG titres significantly decreased, remaining however 10 times higher compared to titres at t0.

**Figure 3:**
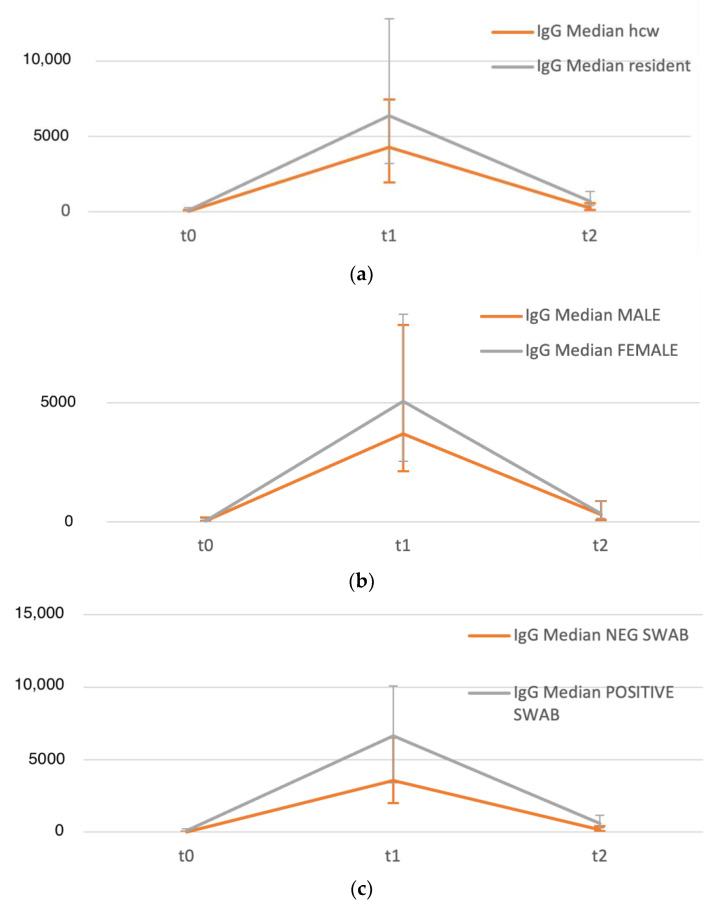
Serodynamics of anti-SARS-CoV-2 IgG according to participant group (t0: N = 534, t1: N = 404, t2: N = 314). (**a**) HCWs vs. residents; (**b**) males vs. females; (**c**) previous SARS-CoV-2 infection vs. no prior infection.

Table 2 reports IgG titres at the three time points among all participants and among participants divided into groups according to participant category, gender, age, and previous SARS-CoV-2 infection. Residents showed higher IgG titres than HCWs at each time point (*p* < 0.001, Figure 3a). No significant differences in antibody titre were found stratifying participants according to gender (Figure 3b); however, statistically significant differences were found at all time points stratifying participants according to age (<75 years vs. ≥75 years), with higher titres among older participants. 

Regarding previous infection, results of Friedman’s tests performed to evaluate trends in titers among participants with a previous positive swab and in participants without previous infection showed a statistical significance (both *p*< 0.001). Post hoc tests were then performed (Wilcoxon rank sum test) to compare, at each time point, the IgG titers of the group of subjects with a prior infection and the group of subjects without previous infection. At all three time points, there was a statistically significant difference between previously positive participants compared to naïve participants, with higher titres among previously infected participants (Figure 3c). 

Furthermore, a significant difference at t0 and t2 (both *p* < 0.001) was found comparing titres among participants with a more recent infection (under 3 months between positive RT-PCR and vaccination) compared to participants with an earlier infection (over 3 months between positive RT-PCR and vaccination). At t0, higher titres were found among participants with a more recent infection, whereas, at t2, higher titres were found among participants with an earlier infection. No significant difference was found among participants with earlier vs. more recent infections at t1 (*p* = 0.273). 

Results of the multivariate analyses are presented in Table 3 and Table 4. After adjusting for age, gender, and participant type, having been previously infected with SARS-CoV-2 was identified as a significant predictor at both time points: two weeks following vaccination (at t1), the Odds of developing a response over threshold was 9.3 (95% Confidence interval, CI 1.07–18.13) in participants with a previous infection compared to naïve participants (*p* 0.044), whereas the Odds of maintaining a response over threshold nine months following vaccination (at t2) was 18.1 (95% CI 7.1–46) in participants with a previous infection compared to naïve participants (*p* < 0.001). The effect of the other evaluated parameters did not reach statistical significance at multivariate analysis.

## 4. Discussion

Long-term follow-up serological studies are essential to characterize the immune response to infection and vaccination against SARS-CoV-2 [20]. Currently available mRNA vaccines generate an anti-spike antibody response, which has been associated with neutralizing activity, and by extension could correlate with protection from infection [21]. Assessing antibody dynamics over time, evaluating differences among individuals based on specific characteristics, and associating these elements with protection from infection and its duration could provide answers to key questions and help inform vaccine strategies, in particular those targeted towards identifying non-responders and individuals that would benefit the most from additional doses [21,22]. Furthermore, serological studies are important to monitor disease transmission in specific populations and can provide information on pandemic progression.

This study reported data from a multicentric, prospective cohort study designed to evaluate SARS-CoV-2 specific IgG titres prior to and following vaccination with Pfizer/BNT162b2 SARS-CoV-2 mRNA vaccine, among 534 residents and HCWs of 13 LTCFs in northwestern Italy. Vaccination elicited a significant neutralizing antibody response considering all participants, with anti-S1 IgG titres two weeks following vaccination significantly higher than titres prior to vaccination. Nine months after vaccination, seropositivity persisted in over 95% of individuals, as other studies have shown [20,23,24]. Comparing titres two weeks vs. and nine months after vaccination, there was a significant decrease by over 93%; however, titres measured at nine months remained ten times higher than titres prior to vaccination, in line with previous reports [12,25,26].

As humoral immunity is only part of the adaptive immune response, a decline in antibody titres does not necessarily translate into reduced protection against infection [20]. Infections in previously exposed individuals (both to vaccination and infection) have been documented [27,28]. In this study, no breakthrough infection was reported between vaccination and the final blood draw, supporting the long-term effectiveness of the immune response generated by Pfizer/BNT162b2 in both naïve and convalescent individuals. It must, however, be noted that the follow-up period of this study coincided with a period of relatively low SARS-CoV-2 incidence between the third and fourth pandemic waves, and prior to the spread of the B.1.617.2 variant in Italy (first identified in the country on 27 November 2021) [29,30].

In this study, significant differences in antibody responses at all three time points were identified among residents vs. HCWs, older vs. younger participants, and previously exposed vs. naïve participants. No significant differences in antibody titre were found stratifying participants according to gender; however, it must be noted that our sample comprised almost 80% of female participants. Other studies have identified gender-based differences in immune responses, which could be determined by multiple factors such as sex steroid concentrations, transcriptional factors, and the partial inactivation of immunoregulatory genes [23]; however, the relationship between sex and humoral response to vaccination is not completely consensually described, particularly in geriatric populations [31,32].

Surprisingly, in our study, higher titres were found among participants ≥75 years compared to under 75 years, and among residents compared to HCWs, contrary to previous studies, which have identified a negative correlation between anti-S titres and age [24,31]. Not only were humoral responses to vaccination significantly higher, but we did not identify more rapid waning of antibodies among elderly participants. It must be noted that our preceding analysis indicated higher exposure levels among residents [15], which is supported by the higher titres measured prior to vaccination, and that previous reports have identified an inconsistently stronger humoral response to infection in older compared to younger individuals [24]. In any case, in multivariate analysis, the influence of age and participant type (resident vs. HCW) did not maintain statistical significance after adjusting for previous infection status and gender.

Concerning the effect of exposure to infection on humoral response, significantly higher titres among previously infected compared to naïve participants were consistently found in our study, in line with general consensus [18,20,25,33]. It has been suggested that cellular immunity, which appears to persist over time, plays a key role in determining this increased response to SARS-CoV-2 antigens [18,33]. Vaccine strategies implemented in several countries, including Italy, have considered hybrid immunity equivalent to receiving a booster dose of vaccine [20,25,28].

Prior to vaccination, significantly higher titres were found among participants with a more recent infection (under 3 months between positive RT-PCR and vaccination), whereas, nine months following vaccination, significantly higher titres were found among participants with an earlier infection (more than 3 months between positive RT-PCR and vaccination). No significant difference was found among participants with earlier vs. more recent infections two weeks after vaccination, suggesting that vaccination elicits a strong response regardless of the timing of previous exposure.

Determining what anti-S antibody levels are sufficient to prevent SARS-CoV-2 infection is a key unanswered question of clinical relevance, and currently there is a lack of consensus on immune correlates of protection [26,28]. Dimeglio et al. have suggested a threshold value for neutralizing antibodies of 141 BAU/mL, which was associated with a level of protection of 89.3% [34]. In this study, we used the higher threshold proposed by Van Praet et al. of 149.1 BAU/mL, and investigated several host factors as potential predictors of developing and maintaining a response over threshold up to 9 months following vaccination. Two weeks after vaccination, a titre over threshold was measured in almost all study participants, whereas approximately only one third maintained a response over threshold after nine months. Previous infection was the only significant predictor of developing and maintaining a response over threshold in both models, in line with previous findings [21,26,31].

This study had several limitations that should be considered. Due to the observational design, findings of this study could be related to differences in demographic and clinical characteristics between groups. We did not record comorbidities, lifestyle and health status characteristics that would have given insight into the impact of those potential factors on titers. A high percentage of participants were female, which may not reflect the intended eligible population. Second, our cohort was relatively small in size, and substantial loss to follow-up occurred between the first and last blood draw, which was mainly due to geriatric residents deceasing in the considered time frame. This is unfortunately an inherent challenge in carrying out longitudinal cohort studies in ageing populations. Third, a single assay was used to quantify antibody levels, and no assessment of T cell modulation was performed. Furthermore, we did not perform multi-omic serum analyses to investigate the immunological differences between prior-infected vs. non-infected individuals that could have led to their differential response to vaccination, which is an important topic that should be further investigated [35,36,37,38]. Assessment of re-infection was based on LTCF records, as we did not plan a systematic follow-up with RT-PCR. Finally, our results could be time-sensitive as this study was conducted prior to the circulation of the B.1.617.2 variant.

## 5. Conclusions

This study evaluated antibody responses up to 9 months from the completion of a primary vaccination cycle with two doses of Pfizer/BNT162b2 SARS-CoV-2 mRNA vaccine. Several countries have now completed the vaccination campaign with the third dose (booster), and others with the fourth dose (second booster). Our study provides information on the antibody response to a primary cycle and the duration of this protection over time. A similar pattern in terms of response and antibody persistence following booster doses (third and fourth) has been described [38]. Several recent studies have focused on the antibody response induced by the third and fourth dose of vaccine against Omicron variants [39,40]. Third-dose boosters have been shown to increase humoral and cellular immunity and provide more short-term protection against symptomatic infection with variants of concern, including Omicron, compared to a two-dose schedule [39]. However, recent data suggest that the protection against symptomatic infection caused by novel variants declines rapidly after the third and fourth doses [39].

In conclusion, despite its limitations, results of this study provided further insights on the humoral response elicited by Pfizer/BNT162b2 SARS-CoV-2 mRNA vaccine, and on host factors determining variations in its magnitude and kinetics. Pre-existing immunity was the strongest predictor of post-vaccination response in both the short and longer term. Results of this study support the strategy of differential provision of booster doses on the basis of exposure status. Further studies are needed to establish immune correlates of protection, particularly in light of the emergence of variants of concern.

## Figures and Tables

**Table 1:** Characteristics of healthcare workers (HCWs) and residents of 13 long-term care facilities participating in the study, Piedmont, Italy, 2021 (t0: N = 534, t1: N = 404, t2: N = 314).

Characteristic	Value
Age at enrollment, median (IQR)	53 (43–78)
Female gender, N (%) ^a^	422 (79.03)
SARS-CoV-2 infection prior to t0, N (%) ^a^	277 (51.87)
Days between positive RT-PCR and t0, median (IQR) t1, median (IQR) t2, median (IQR)	70.5 (47.5–262) 112 (80.5–302) 355 (331–531)
Seropositivity for IgG anti-SARS-CoV-2, N (%) ^b^ t0 t1 t2	202 (37.83) 402 (99.5) 297 (94.59)
IgG response over threshold, N (%) ^b^ t0 t1 t2	135 (25.28) 396 (98) 85 (27.07)
Days between second vaccine dose and t1, median (IQR) t2, median (IQR)	14 (14–19) 250 (250–279)

^a^ percentage over total at t0; ^b^ percentage over total at each respective time point.

**Table 2:** Anti-SARS-CoV-2 IgG titres (BAU/mL) of study participants, overall and stratified according to groups, Piedmont, Italy, 2021 (t0: N = 534, t1: N = 404, t2: N = 314).

Groups	t0 Titre, Median (IQR) [N534]	t1 Titre, Median (IQR) [N 404]	t2 Titre, Median (IQR) [N 314]
Participant category: HCWs Residents *p*-value ^a^	19.9 (0–109.7) [N 369] 73.5 (16.5–252.3) [N 165] <0.001	4266.8 (2328.7–7435.5) [N 258] 6377.5 (3177.3–12,806.4) [N 146] <0.001	230.3 (113.3–570.4) [N 195] 677.1 (264–1339) [N 119] <0.001
Gender: Female Male *p*-value ^a^	38 (0–136.4) [N 422] 38.8 (0–181.7) [N 112] 0.488	5061.3 (2528.8–8713.2) [N 315] 3690 (2128.9–8272.3) [N 89] 0.116	336.7 (149.7–856.3) [N 252] 293.7 (66.1–874.5) [N 62] 0.150
Age: <75 years ≥75 years *p*-value ^a^	30.3 (0–124.8) [N 389] 64 (15.4–233.5) [N 145] <0.001	4250.6 (2380.8–7515.8) [N 275] 7101.7 (3356–12,806.4) [N 129] <0.001	267.4 (117.2–637.1) [N 208] 709.1 (237.5–1208.2) [N 106] <0.001
Previous SARS-CoV-2 infection: No previous infection; Previous infection, >3 months from positive RT-PCR to vaccination; Previous infection, ≤3 months from positive RT-PCR to vaccination *p*-value (no previous infection vs. previous infection) ^a^	0 (0–32.9) [N 313] 53.2 (20.6–166.4) [N 92] 136.4 (53.3–384) [N 129] <0.001	3568.1 (2006.8–6522.1) [N 199] 7297 (3658.2–11,379.5) [N 86] 6267.5 (3345.3–9852.4) [N 119] <0.001	177.8 (66.4–422.7) [N 166] 1079.2 (438.8–2176.4) [N 49] 502.7 (271.6–897.7) [N 99] <0.001
All participants	36.8 (0–151.8)	4791.3 (2494.6–8634.4)	316.4 (127.7–872.1)

HCWs: healthcare workers; IQR: interquartile range; ^a^ difference among groups at each time point at Mann–Whitney U tests.

**Table 3:** Predictors of developing a significant antibody response two weeks after a full vaccination cycle with Pfizer/BNT162b2 SARS-CoV-2 mRNA vaccine among healthcare workers (HCWs) and residents of 13 long-term care facilities of Piedmont, Italy, 2021.

	OR (95% CI)	*p*-Value
Residents HCWs	Ref 0.73 (0.02–23.68)	0.859
Age	0.84 (0.86–1.04)	0.218
Male gender Female gender	Ref 2.64 (0.54–12.85)	0.228
No prior SARS-CoV-2 infection Previous SARS-CoV-2 infection confirmed by RT-PCR	Ref 9.3 (1.07–18.13)	0.044

CI: confidence interval; OR: Odds ratio; RT-PCR: reverse-transcription polymerase chain reaction.

**Table 4:** Predictors of maintaining a significant antibody response 9 months after a full vaccination cycle with Pfizer/BNT162b2 SARS-CoV-2 mRNA vaccine among healthcare workers (HCWs) and residents of 13 long-term care facilities of Piedmont, Italy, 2021.

	OR (95% CI)	*p*-Value
Residents HCWs	Ref 0.47 (0.09–2.36)	0.358
Age	0.97 (0.94–1.01)	0.105
Male gender Female gender	Ref 1.01 (0.42–2.41)	0.991
No prior SARS-CoV-2 infection Previous SARS-CoV-2 infection confirmed by RT-PCR	Ref 18.1 (7.1–46)	<0.001

CI: confidence interval; OR: Odds ratio; RT-PCR: reverse-transcription polymerase chain reaction.

## Data Availability

Data will be made available upon reasonable request.
